# Paraptosis‐related genes regulate tumor immune microenvironment and predict prognosis in breast cancer

**DOI:** 10.1002/ccs3.70056

**Published:** 2025-12-08

**Authors:** Ziyi Dong, Yanfang Yang, Mingyu Zhu, Hui Liu, Yaoyang Guo, Haiyang Zhang, Zhansheng Jiang

**Affiliations:** ^1^ Department of Integrative Oncology Department of the Second Breast Cancer Tianjin Medical University Cancer Institute and Hospital National Clinical Research Center for Cancer Key Laboratory of Cancer Prevention and Therapy Tianjin's Clinical Research Center for Cancer Tianjin Cancer Hospital Airport Hospital Tianjin Medical University Tianjin China; ^2^ Tianjin Institute of Coloproctology The Institute of Translational Medicine Tianjin Union Medical Center Nankai University Tianjin China

**Keywords:** breast cancer, drug sensitivity, paraptosis, *PI4KB*, tumor microenvironment

## Abstract

Paraptosis is a non‐apoptotic form of programmed cell death, distinct from classical apoptosis in morphology and mechanism. It has been implicated in tumor resistance and immune microenvironment remodeling, but its role in breast cancer (BC) remains unclear. We classified patients into two subtypes based on the expression of paraptosis‐related genes. Then, we systematically analyzed the prognosis and tumor microenvironment (TME) associated with these subtypes. In addition, we developed a risk score, named the paraptosis‐related risk score (PRRS). We comprehensively analyzed the correlation of paraptosis with BC prognosis, TME, immune score, and drug sensitivity. Then, we performed in vitro experiments to verify the effect of PI4KB on BC. The PRRS can effectively predict the prognosis and immunity of BC. Low PRRS was associated with a favorable prognosis, characterized by reduced tumor purity and enhanced immune cell infiltration. In addition, PRRS can help identify patients who are suitable for specific drug therapies. Finally, we found that PI4KB was highly expressed in BC. Knockdown of PI4KB expression significantly suppressed BC cell proliferation and migration. Our study establishes a robust framework for BC subtype classification and prognostic prediction, providing novel guidance for personalized therapeutic strategies.

## Graphical abstract

Graphical abstract illustrating the role of paraptosis‐related genes in shaping the tumor immune microenvironment and predicting clinical outcomes in breast cancer.

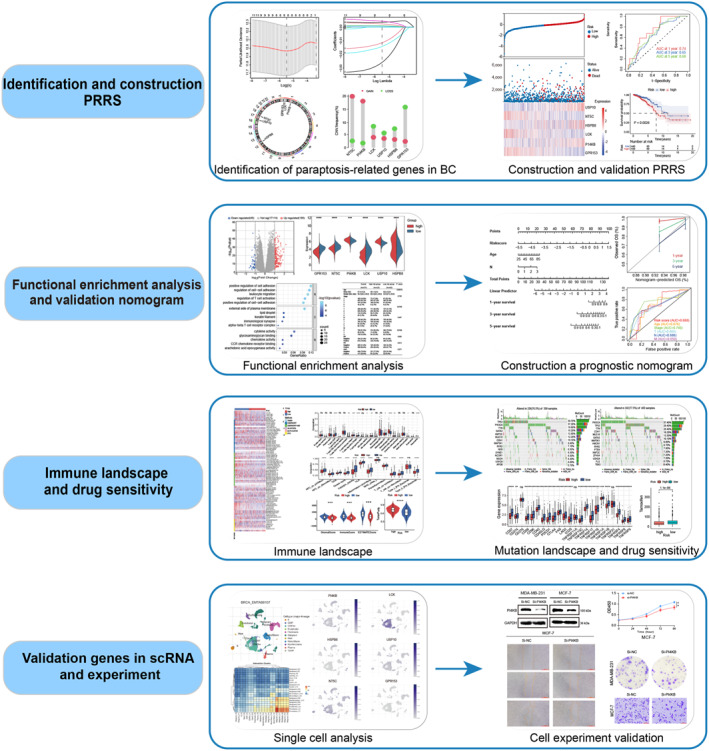

## 1 INTRODUCTION

Breast cancer (BC) is one of the most common malignant tumors in women and has become a major disease threatening women's health worldwide.^1^ The treatment of BC has gradually developed into a multidisciplinary treatment model, including surgery, chemotherapy, endocrine therapy, targeted therapy, and radiation therapy. Most patients with early‐stage BC have a good prognosis.^2^ However, some patients remain at risk for tumor recurrence and distant metastasis.^3^ Despite advances in therapeutic approaches, current treatment strategies often fail to achieve satisfactory disease control in patients with advanced BC or those with specific pathological subtypes, such as triple‐negative breast cancer (TNBC) and HER2‐positive BC. Immunotherapy is currently mainly used in TNBC, but its application in other subtypes still requires further exploration.^4^, ^5^ Therefore, the early identification and validation of reliable and accurate biomarkers for the effective prediction and personalized treatment of BC remain critical challenges in current clinical research.^6^

Paraptosis represents a nonapoptotic form of programmed cell death that is morphologically and mechanistically distinct from classical apoptosis.^7^, ^8^ It is characterized mainly by massive vacuolization of the endoplasmic reticulum (ER) and mitochondria, without caspase activation and apoptotic body formation.^9^ The induction of paraptosis is associated with multiple signaling pathways, mainly involving key processes such as ER stress, imbalance of calcium homeostasis, and protein aggregation.^9^, ^10^ A key feature of paraptosis is the formation of extensive cytoplasmic vacuoles, primarily triggered by ER stress. This stress activates the unfolded protein response (UPR) when the ER fails to properly fold proteins or when calcium homeostasis is disrupted, ultimately leading to vacuolization and cell death.^11^, ^12^ The transfer of calcium between the ER and mitochondria plays a critical role in the initiation of paraptosis.^13^ Sustained calcium influx leads to loss of mitochondrial membrane potential and generation of reactive oxygen species (ROS), which further exacerbates cell damage and death.^14^ Abnormal protein aggregation and activation of the mitogen‐activated protein kinase (MAPK) signaling pathway are mechanistically involved in the induction and execution of paraptosis.^15^, ^16^ For example, long‐lasting activation of extracellular regulated kinase 1/2 (ERK1/2) has been shown to promote paraptosis and trigger vacuolization by regulating protein synthesis and cytoskeleton remodeling.^17^

In the era of precision medicine, growing attention has been directed toward the potential role of paraptosis in cancer therapy.^9^, ^18^ In contrast to conventional apoptotic pathways, paraptosis operates independently of caspase activation. This caspase‐independent nature renders paraptosis a promising alternative mechanism for the development of anti‐tumor therapies.^19^ For example, in the treatment of BC, TNBC has a limited therapeutic effect due to high resistance to traditional treatment. By inducing ER stress and disrupting calcium homeostasis, paraptosis inducers can effectively trigger cell death in TNBC cells and help overcome drug resistance.^20^ Similarly, HER2‐positive BC also shows a high sensitivity to the induction of paraptosis, further demonstrating that paraptosis can be used as an effective therapeutic strategy.^21^ Paraptosis has been shown to modulate the TME and influence immune cell function.^22^, ^23^, ^24^ However, its precise role in immunotherapy and the regulation of cellular immune mechanisms remains further study.^9^, ^12^, ^25^ In addition, a variety of compounds can effectively induce paraptosis to exert anti‐tumor effects.^26^, ^27^ By inducing ER stress and mitochondrial dysfunction, the natural compound curcumin inhibits tumor cell proliferation through the activation of paraptotic pathways.^28^, ^29^, ^30^

As mentioned earlier, paraptosis plays an important role in inhibiting tumorigenesis and progression. Therefore, we developed a predictive model for BC based on the expression of genes involved in paraptosis. In addition, we investigated the biological processes (BPs), pathways, and immune infiltration status associated with paraptosis‐related genes. Meanwhile, we further focused on *PI4KB* and explored its function in BC. Our study highlights the clinical significance of paraptosis‐related genes in BC development, offering important implications for prognosis prediction and the identification of novel therapeutic approaches.

## 2 MATERIALS AND METHODS

### 2.1 Data collection

RNA sequencing (RNA‐seq) for BC and normal tissues were obtained from the TCGA database (https://www.cancer.gov/tcga).^31^ Gene expression profiles in TPM format (transcripts per million) were used, ensuring normalization for both gene length and sequencing coverage. Samples lacking complete clinical annotations or with survival duration under 30 days were excluded from analysis. Clinical data were obtained, including age; TNM stage; expression status of ER, PR, and HER2, survival time; and status. To validate the prognostic relevance of paraptosis, an external dataset (GSE20711) from the GEO database was utilized.^32^ Genes associated with paraptosis were identified through the Molecular Signatures Database (MsigDB) and published studies (Table S1).^33^ Single‐cell RNA sequencing (scRNA‐seq) profiles from the EMTAB8107 dataset were sourced using the TISCH2 platform.^34^

### 2.2 Development of paraptosis‐related prognosis signature

Selected paraptosis‐related genes were screened by univariate Cox regression, least absolute shrinkage and selection operator (LASSO) regression and multivariable Cox regression analysis to establish a new prognostic gene‐based risk model.^35^ Each sample's risk score was calculated using the following formula:

$$\text{Risk}\;\text{score}=\Sigma \text{expgen}e_{i}\times \beta_{i}$$

where expgene_*i*_, and *β*_*i*_ corresponds to both the expression value of the gene and the associated coefficient derived from the LASSO model. Across all datasets, participants were stratified into high‐ and low‐risk categories using the median risk score as the threshold. To analyze the performance of the prognosis signature, Kaplan–Meier survival curves were generated using the “survival” and “survminer” packages in R. We assessed the robustness of the prognostic model using time‐dependent receiver operating characteristic (ROC) analysis implemented using the “timeROC” R package.

### 2.3 Identification and enrichment analysis of the differentially expressed genes

The “limma” R package was employed to identify differentially expressed genes (DEGs) by comparing expression profiles between the high‐ and low‐risk groups. Genes with expression levels showing |log2 fold change (FC)| >1 and adjusted *p*‐values <0.01 were included. Subsequently, functional and pathway enrichment analyses based on Gene Ontology (GO) and Kyoto Encyclopedia of Genes and Genomes (KEGG) databases were performed using the “clusterProfiler” package in R.^36^

### 2.4 Construction of the paraptosis‐related nomogram

Both univariate and multivariate Cox regression analyses were applied to paraptosis‐associated genes in combination with relevant clinical parameters. A prognostic nomogram was constructed using these independent variables to estimate BC survival probabilities at 1, 3, and 5 years.

### 2.5 Immune infiltration landscape

CIBERSORT (http://cibersort.stanford.edu/) was utilized to estimate the abundance of 22 tumor‐infiltrating immune cells (TIICs) subtypes per sample.^37^ Single sample Gene Set Enrichment Analysis (ssGSEA) (http://www.gsea‐msigdb.org/gsea/index.jsp) was applied to predict the abundance of 28 TIICs in individual tissue samples.^38^ Furthermore, we used the R package “estimate” to calculate each sample's ESTIMATE score.^39^ The tumor immune exclusion score was calculated by the tumor immune dysfunction and exclusion (TIDE) (http://tide.dfci.harvard.edu) analysis.^40^ In addition, we analyzed differential expression of the human leukocyte antigen (HLA) family genes and key co‐stimulatory molecules between high‐ and low‐risk groups.

### 2.6 Tumor mutational analysis

Genomic mutation data for BC were retrieved from the cBioPortal repository (https://www.cbioportal.org/). We utilized the “maftools” package in R (v 4.3.1) to visualize the mutation profiles of BC cases.^41^

### 2.7 Drug sensitivity analysis

Half‐maximal inhibitory concentration (IC50) values for standard chemotherapeutics were estimated using RNA‐seq data using the “oncoPredict” R package.^42^ Differences in drug sensitivity across risk groups were assessed using the Wilcoxon rank‐sum method.

### 2.8 Validation of the paraptosis‐related genes using scRNA‐seq analysis

To identify cellular heterogeneity and map signature gene expression, we employed the EMTAB8107 scRNA‐seq dataset obtained from the TISCH2 resource.^34^ Cell populations were annotated by examining marker gene expression profiles, enabling classification into distinct cell types. We applied uniform manifold approximation and projection (UMAP) for cell clustering to depict spatial patterns of signature gene expression.

### 2.9 Cell lines, cell culture, and cell transfection

We used the following human cell lines in this study: MDA‐MB‐231 (Homo sapiens, female, breast adenocarcinoma; pleural effusion), official name MDA‐MB‐231, RRID: CVCL_0062, and MCF‐7 (Homo sapiens, female, breast adenocarcinoma; mammary gland/pleural effusion), official name MCF‐7, RRID: CVCL_0031. Cell lines were obtained from Procell Life Science & Technology Co., Ltd. (Wuhan, China; CL‐0150 for MDA‐MB‐231], [CL‐0149 for MCF‐7]) in November 2024. Cells were authenticated by the supplier (Procell) using STR profiling and confirmed to be correctly identified. Cells were confirmed to be free from *mycoplasma* contamination prior to the experiments. MDA‐MB‐231 and MCF‐7 cells were cultured in RPMI‐1640 medium (Hyclone, Logan, UT, USA). All media were supplemented with 10% fetal bovine serum (FBS, Gibco, Carlsbad, CA, USA) and 1% penicillin‐streptomycin. Cells were cultured at 37°C and 5% CO2. Transient small interfering RNA (siRNA) transfection was used to knock down *PI4KB*, with transfection reagents purchased from Yeasen (Shanghai, China). The siRNA oligonucleotides were synthesized by the Public Protein/Plasmid Library. (Jiangsu, China).

### 2.10 Western blotting

Total proteins in cell lysates were extracted with RIPA lysate (Solarbio, Beijing, China), protease inhibitors (Biotek, China) were added to prevent protein degradation, and the concentration of proteins was quantified using a BCA protein assay kit. Protein extracts were then resolved using 10% SDS‐polyacrylamide gel electrophoresis followed by transfer onto PVDF membranes. The PVDF membranes were blocked in TBST with 5% nonfat dry milk for 1 h at room temperature. Membranes were then probed overnight at 4°C with antibodies against PI4KB (Proteintech, 13247‐1‐AP) and GAPDH (Proteintech, 60004‐1‐Ig). HRP‐conjugated secondary antibodies were applied for 1 h at room temperature. Finally, protein bands were visualized by chemiluminescent reagents.

### 2.11 Cell viability assay (cell counting Kit‐8, CCK‐8)

Approximately 2000 cells were seeded into each well of a 96‐well plate and cultured for 0, 24, 48, 72, and 96 h. Subsequently, 10% CCK‐8 reagent (Biosharp, Beijing) was introduced to each well. After 2 h of incubation, the absorbance (OD) of each well was detected at 450 nm with an enzyme labeler.

### 2.12 Colony formation assay

A total of 300 tumor cells were plated into each well of a 6‐well plate and maintained at 37°C in a 5% CO_2_ incubator. After 14 days, colonies were fixed with 4% paraformaldehyde for 15 min, then stained with 0.1% crystal violet (#G1063, Solarbio) for an additional 15 min. The cells were then washed three times with PBS and colonies were counted.

### 2.13 Transwell assay

Tumor cells were resuspended in serum‐free medium at a concentration of 1.2 × 10^5^ cells/mL, and 200 μL of this suspension was added to the upper compartment of a Transwell insert (FALCON, Shanghai, China). Complete culture medium (600 μL) was placed in the lower well to serve as a chemoattractant. 24 h later, cells were fixed with 4% paraformaldehyde for 20 min, and then stained with 0.1% crystal violet (#G1063 Solarbio) staining solution for 20 min. The number of migrated cells was observed and counted under a light microscope.

### 2.14 Wound healing assay

After ensuring that the cell density is 90% or greater confluence, a sterile 200 μL pipette tip was employed to draw a straight scratch vertically across the cell monolayer. The plate was then rinsed three times with PBS. Fresh serum‐free medium was added, and the initial wound width at time 0 was documented. Cells were maintained under standard incubation conditions (37°C, 5% CO_2_) and the scratches were imaged again after 24 and 48 h, respectively.

### 2.15 Statistical analyses

Statistical analysis and plotting were performed using R version 4.3.1 and GraphPad Prism 9. Results were reported as mean ± SD, and group differences were assessed using either Student's *t*‐test or the Wilcoxon rank‐sum test. For comparisons involving more than two groups, either one‐way ANOVA or the Kruskal–Wallis test was applied as appropriate. Survival curves were evaluated using the log‐rank test, with a significance threshold set at *p* < 0.05.

## 3 RESULTS

### 3.1 Identification of paraptosis‐related genes in BC

**FIGURE 1:**
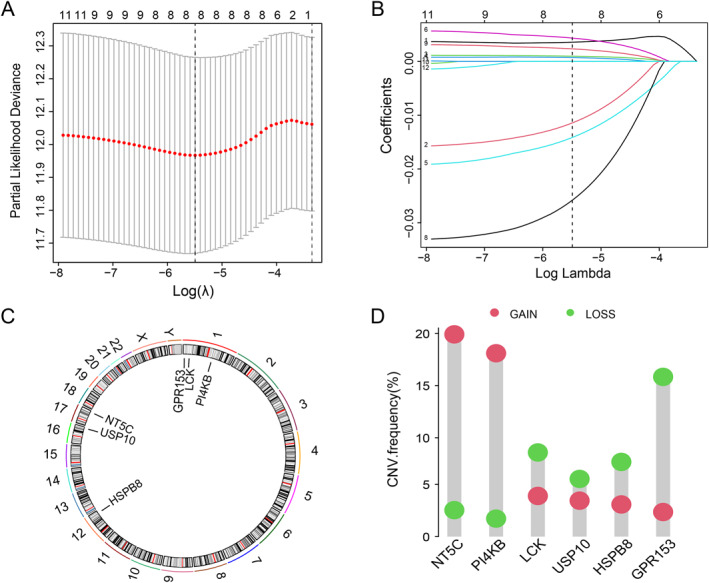
Identification of paraptosis‐related genes. (A, B) LASSO regression of the twelve OS‐related genes. (C) Location of the six‐gene signatures in the chromosome. (D) The copy number variation status of the six key genes.

The graphical abstract presents an integrated flowchart summarizing the overall study design. To investigate the predictive value of paraptosis, we analyzed paraptosis‐related genes with univariate Cox regression model from the TCGA‐BRCA training cohort and obtained 12 genes that significantly affected overall survival (OS) of patients (Table S2). Subsequently, LASSO regression analysis was performed on these genes and then eight genes with nonzero coefficients were identified (Figure 1A,B). Ultimately, multivariate Cox analysis identified six key genes that could be retained for constructing a paraptosis‐related risk score (PRRS) in BC (Table S3). Figure 1C shows the chromosomal locations of these six key genes. Then we assessed BC samples for copy number variation (CNV) in these six genes, a genomic variant that may significantly affect gene expression.^43^ We found that CNV was prevalent in six genes. Among these, *NT5C* and *PI4KB* showed relatively high levels of amplification, whereas *USP10*, *HSPB8*, *LCK*, and *GPR153* showed copy number losses (Figure 1D).

### 3.2 Construction and validation of PRRS

**FIGURE 2:**
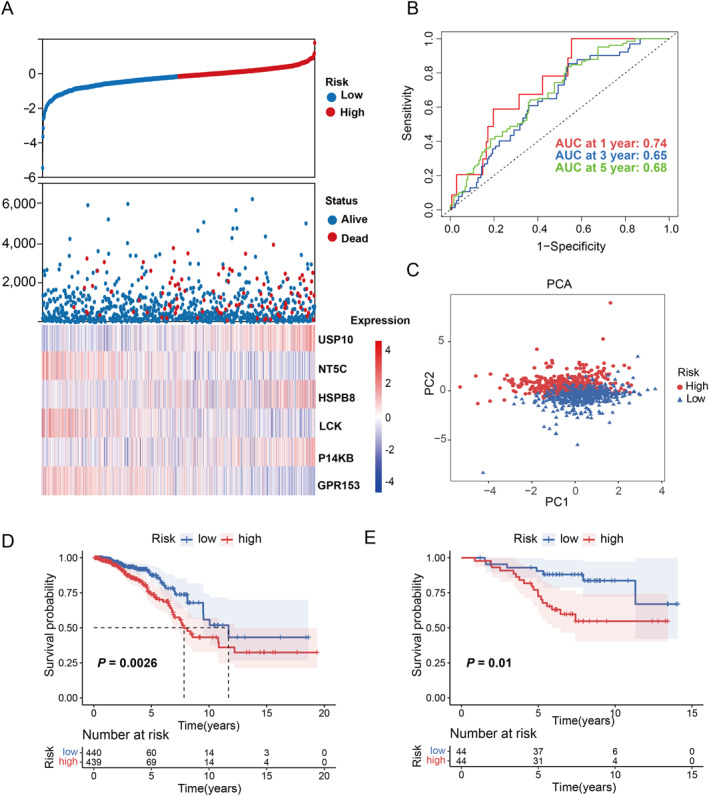
Construction and verification of the prognostic model. (A) Risk curve of PRRS and expression heatmap of model genes. (B) Receiver operating characteristic curves of the PRRS. (C) PCA analysis of the two subgroups. (D) KM curve analysis of differences in OS between two subgroups in TCGA cohort. (E) KM curve analysis of differences in OS between two subgroups in GEO cohort. OS, overall survival; PRRS, paraptosis‐related risk score.

**FIGURE 3:**
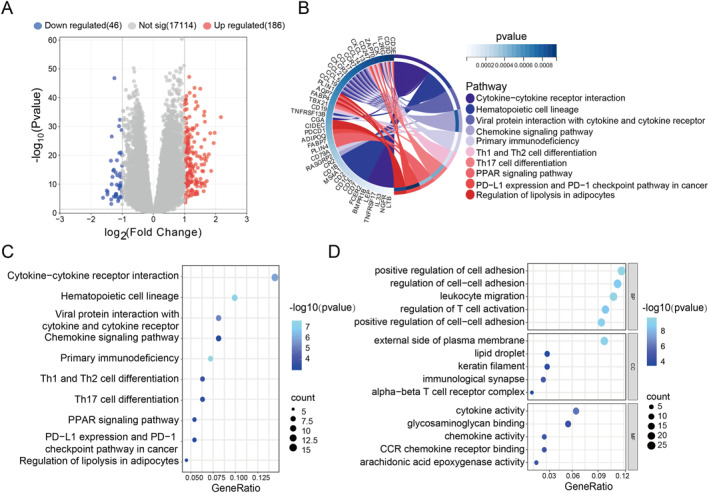
Functional enrichment analysis of the DEGs in PRRS groups. (A) Volcano plot of DEGs based on PRRS. (B) Circle map of KEGG enrichment analysis. (C) Dot plot of KEGG enrichment analysis. (D) Dot plot of gene ontology enrichment analysis. DEGs, differentially expressed genes; KEGG, Kyoto encyclopedia of genes and genomes; PRRS, paraptosis‐related risk score.

We verified the predictive value of the above key paraptosis‐related genes by calculating risk scores based on gene expression levels. LASSO regression coefficients for the six genes using the following formula: risk score = *h*_0_ exp [(0.00585764541858519 × *USP10*) + (−0.0187881349300088 × *NT5C*) + (0.00132173365899394 × *HSPB8*) + (−0.019757663 × *LCK*) + (0.00551101330066126 × *PI4KB*) + (−0.0304533563586096 × *GPR153*)]. Patients in the TCGA‐BRCA training cohort were categorized into high‐ and low‐risk groups according to the median risk score. Clinical outcomes and signature gene expression levels for each patient were grouped by risk score (Figure 2A, Figure S1 and Table S4). With AUC values of 0.74, 0.65, and 0.68 at 1‐, 3‐, and 5‐year time points, the gene signature exhibited strong predictive capability for patient survival (Figure 2B). PCA did a good job of distinguishing high‐risk from low‐risk groups (Figure 2C). The survival curves showed that the low‐risk group had a favorable OS compared with the patients in the high‐risk group (*p* = 0.0026, Figure 2D). External validation was performed using the BC cohort from the GEO database to further confirm the prognostic value of PRRS. According to the computed risk score, the cohort of 88 patients was split into high‐risk (*n* = 44) and low‐risk (*n* = 44) groups based on the median threshold. Kaplan–Meier survival analysis demonstrated that patients in the high‐risk group exhibited significantly worse survival compared to the low‐risk group (*p* = 0.01, Figure 3E).

### 3.3 Functional enrichment analysis of the DEGs in high and low risk groups

In this study, we explored the potential biological roles and mechanisms of paraptosis‐associated genes in BC. A functional enrichment assessment was conducted on 233 DEGs identified in subgroups from the TCGA cohort (Figure 3A). KEGG pathway analysis indicated that the DEGs were predominantly involved in cytokine–cytokine receptor interaction, hematopoietic cell lineage, and viral protein interaction. In addition, DEGs were also enriched in T cell regulation and PD1 expression (Figure 3B,C). GO term analysis categorized the DEGs into three major functional groups: BPs, cellular components (CCs), and molecular functions (MFs). Enriched pathways mainly included those underlying positive regulation of cell adhesion, external side of plasma membrane, and regulation of T‐cell cytokine activity (Figure 3D). These results suggest that the identified DEGs contribute to immune signaling and tumor development. Prognostic differences between the high‐ and low‐risk groups may be influenced by immune response activity.

### 3.4 Construction of the paraptosis‐related prognostic nomogram

**FIGURE 4:**
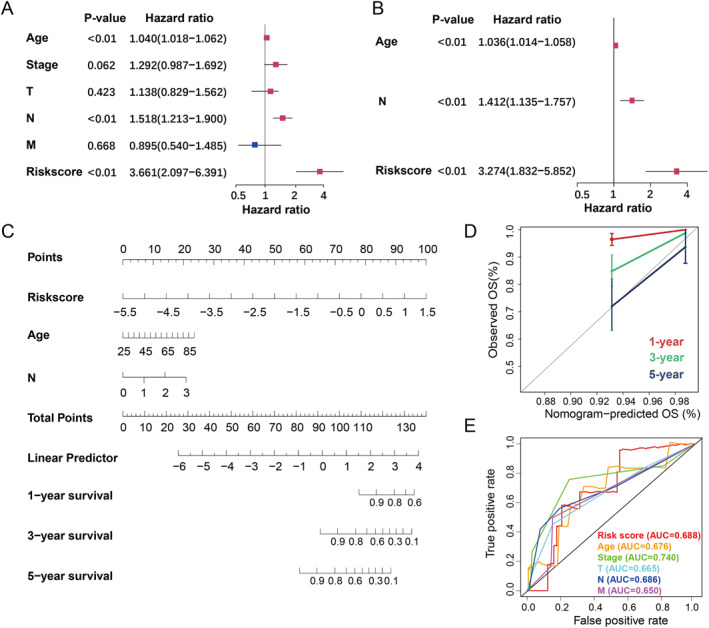
Construction and validation of the nomogram. (A) Univariate regression and (B) multivariate regression of the clinicopathological indicators. (C) A nomogram predicting the OS in breast cancer patients. (D) Calibration curve of the nomogram. (E) Receiver operating characteristic curves of the nomogram predicting OS of patients. OS, overall survival.

To further assess PRRS prognostic relevance, we conducted a stratified analysis across subgroups with varying clinical features. The results indicate that patients classified as low‐risk generally exhibited more favorable clinical outcomes, except for those at the M1 stage. Patients in the low‐risk group showed significantly better OS, especially among older individuals, those with ER+/PR+/HER2− tumor profiles and those diagnosed at an early stage (Figure S2). Subsequently, significant clinicopathological indicators and the gene signatures were subject to univariate and multivariate Cox analyses. Univariate Cox regression revealed significant associations of age [hazard ratio (HR): 1.040, 95% confidence interval (CI): 1.018–1.062, *p* < 0.01], N staging (HR: 1.518, 95% CI: 1.213–1.900, *p* < 0.01), and the PRRS (HR: 3.661, 95% CI: 2.097–6.391, *p* < 0.001) were significantly related to BC prognosis (Figure 4A). Multivariate analysis confirmed that age (HR: 1.036, 95% CI: 1.014–1.058, *p* < 0.01), N staging (HR: 1.412, 95% CI: 1.135–1.757, *p* < 0.01), and the PRRS (HR: 3.274, 95% CI: 1.832–5.852, *p* < 0.01) were still found to be independent prognostic factors (Figure 4B). To enhance prognostic precision in BC, we constructed a nomogram capable of estimating 1‐, 3‐, and 5‐year OS probabilities based on key variables (Figure 4C). Model performance was evaluated using ROC analysis and calibration plots, both of which demonstrated its accuracy and clinical applicability (Figure 4D,E).

### 3.5 PRRS predicts immune landscape and drug sensitivity in BC

**FIGURE 5:**
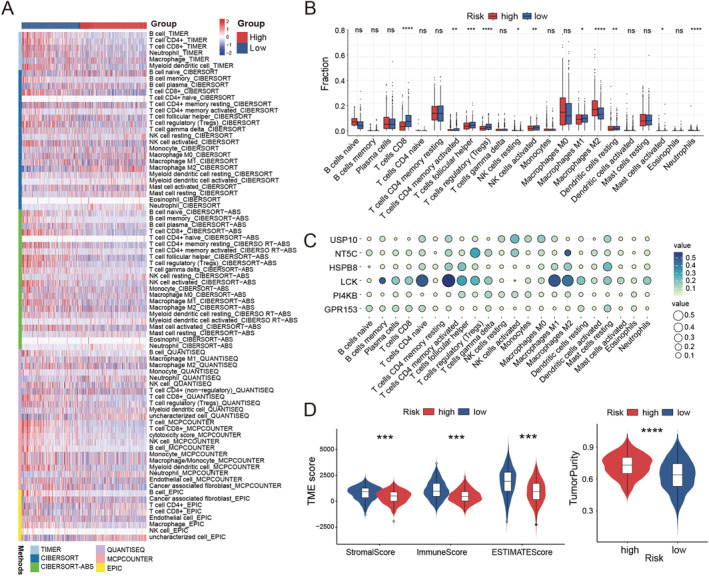
Immune landscape between the two PRRS groups. (A) Different TME evaluation algorithms in high and low risk groups. (B) Proportions of 22 immune cells in the low‐risk and high‐risk groups. (C) Correlation of signature genes and immune cell infiltration in the PRRS model. (D) Correlation between PRRS and TME score. (*p < 0.05; **p < 0.01; ***p < 0.001; ****p < 0.0001; ns, not significant). PRRS, paraptosis‐related risk score.

TME is critically involved in cancer development, therapeutic resistance, and response to immunotherapy.^44^, ^45^ We employed several computational approaches to assess differences in immune cell infiltration between high‐ and low‐risk groups (Figure 5A). Furthermore, we utilized CIBERSORTx to investigate the immune landscape of patients stratified by PRRS risk levels and showed that patients in the high‐risk group had significantly lower numbers of CD8+ T cells, M1 macrophages, and Tregs compared with patients at lower PRRS (Figure 5B). At the same time, significant associations were observed between the expression of six core genes and specific immune cell populations (Figure 5C). We further analyzed the TME in BC patients using the ESTIMATE algorithm. The results demonstrated that the low‐risk group had significantly higher stromal and immune scores compared to the high‐risk group, whereas the high‐risk group exhibited greater tumor purity (Figure 5D). ssGSEA also revealed low expression of most TIICs in the high‐risk group, including activated B cells, CD4+ T cells, CD8+ T cells, and dendritic cells (Figure S3A). We then observed significantly higher levels of inflammation promotion, immune checkpoints, cytolysis, T cell co‐inhibition, T cell co‐stimulation, parainflammation, type 2‐related IFN response, antigen‐presenting cell (APC) co‐inhibition, CCR, and HLA in the low‐risk group (Figure S3B). We also examined immune checkpoints and HLA families in the high‐ and low‐risk groups and showed that immune checkpoints and HLA families were expressed at higher levels in the low‐risk group (Figure S3C, D). These findings imply that individuals classified as low‐risk may respond more favorably to immunotherapeutic strategies.

**FIGURE 6:**
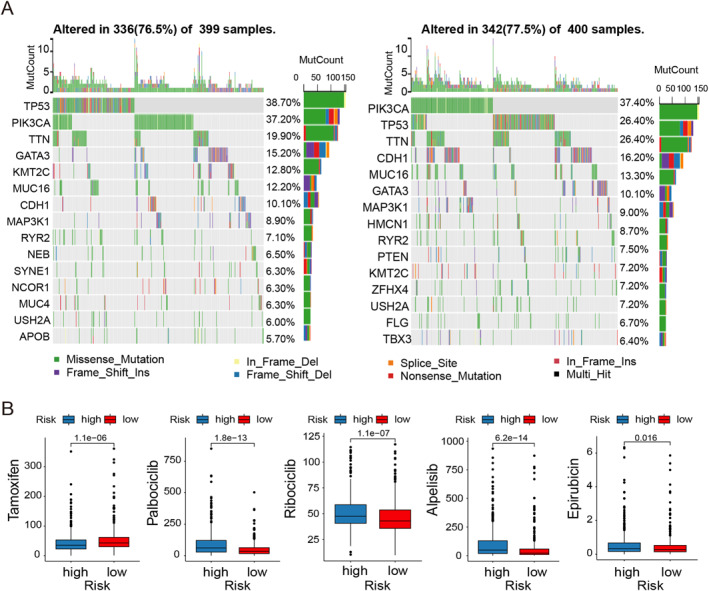
Mutation landscape and drug sensitivity in the PRRS groups. (A) Waterfall plots of somatic mutational signatures of high and low PRRS groups. (B) Correlation between PRRS groups and drug sensitivity. PRRS, paraptosis‐related risk score.

We next profiled the mutational landscape of BC and investigated its association with PRRS. In the high‐risk group, mutations in *TP53*, *PIK3CA*, and *TTN* were found in 38%, 31%, and 20% of patients. In the low‐risk group, mutations in *TP53*, *PIK3CA*, and *TTN* were found in 31%, 39%, and 18% of patients (Figure 6A).

According to the different types of BC, the main treatment methods are also different. To explore the potential role of PRRS in guiding precision medicine for BC, we assessed the clinical utility of the PRRS model by analyzing drug IC50 values for commonly used treatments (Figure 6B). On the basis of the mutational waterfall plot described above, we found that the high‐risk subgroup demonstrated increased susceptibility to the *PIK3CA* inhibitor Alpelisib. Moreover, the high‐risk cohort exhibited elevated IC50 values for Epirubicin and CDK4/6 inhibitors (Palbociclib, Ribociclib), but lower IC50 levels for Tamoxifen. The results revealed that Tamoxifen may be particularly effective in patients within the high‐risk group.

### 3.6 Validation of the paraptosis‐related genes in scRNA‐seq analysis

**FIGURE 7:**
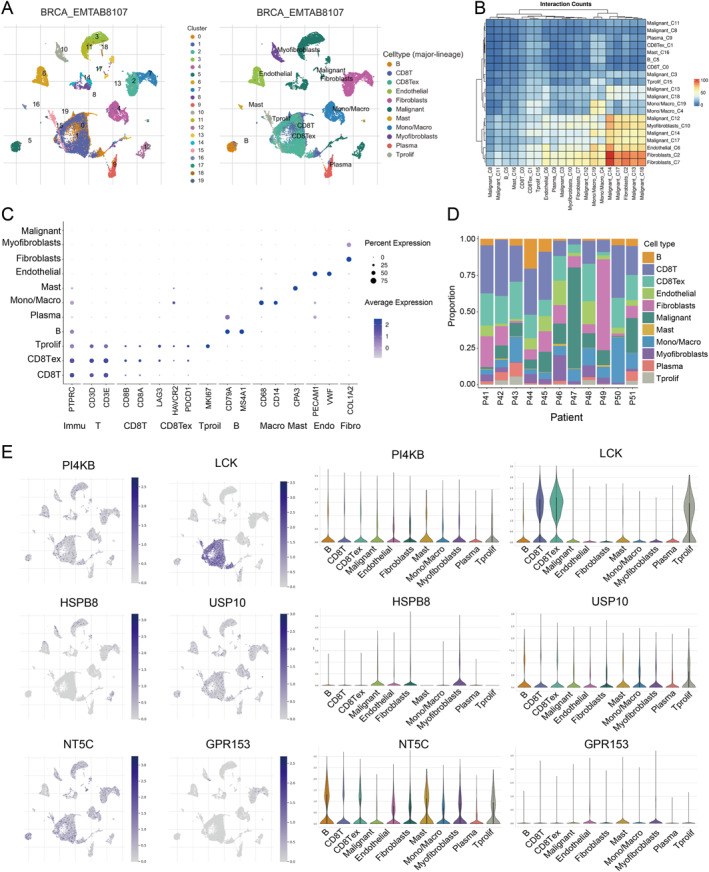
Validation of the six key genes. (A) The UMAP analysis of 10 cellular subtypes in the scRNA‐seq data. (B) Correlation between different cell types. (C) Marker genes for different cell types in breast cancer. (D) Proportional distribution of various cell types within each sample. (E) The expression level of the model genes in each cellular subtype.

This study also included E‐MTAB‐8107 data, which provided high‐quality single‐cell RNA sequencing information for tumor tissue. After quality control, normalization, and PCA‐based dimensionality reduction, all analyzed cells were classified into 11 distinct cell types based on established marker genes and dataset annotations (Figure 7A). We further investigated the correlations among different cell clusters and identified a strong association between fibroblasts and malignant cells, suggesting that targeting fibroblasts may hold prognostic significance (Figure 7B). Marker genes for different cell types, such as COL1A2 for fibroblasts and CD68 and CD14 for macrophage cells, all showed strong expression specificity (Figure 7C). Differences of each sample in cell type distribution can reveal clusters of cells that were highly correlated with disease, and the proportion of different cell types at the sample level was shown in Figure 7D. A UMAP plot was generated to visualize the expression patterns of characteristic genes across the identified cell types (Figure 7E). The results indicated that the six paraptosis‐related genes were differentially expressed among immune cell populations, further highlighting the close association between PRRS and the TME.

### 3.7 Identifying the oncogenic role of PI4KB in BC

**FIGURE 8:**
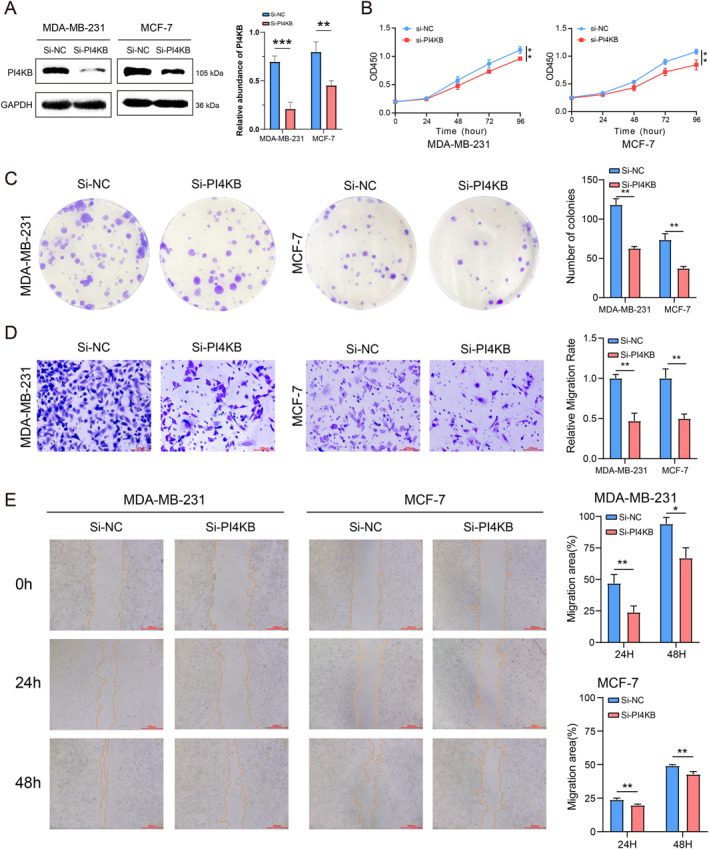
*PI4KB* promotes BC cells proliferation and invasion. (A) The expression of *PI4KB* was knocked down by siRNA. (B, C) CCK‐8 assay and colony formation showed that the proliferative ability of BC cells decreased after knockdown of *PI4KB*. (D, E) Transwell and wound healing experiments showed that the migration ability of BC cells decreased after knocking down *PI4KB*. The above experiments were repeated three times, and the expression of *PI4KB* was analyzed by one‐way ANOVA. Other experiments were analyzed by *t*‐test. *p* < 0.05 was statistically significant. (*p < 0.05; **p < 0.01). BC, breast cancer.

PI4KB was prioritized for experimental validation because it showed strong oncogenic and prognostic relevance and was mechanistically linked to the ER stress–AKT/MAPK axis involved in paraptosis.^46^, ^47^, ^48^ Among the three oncogenic genes identified in the PRRS model, HSPB8 and USP10 have already been functionally characterized in paraptosis‐related pathways, whereas the role of PI4KB remains unexplored, making it a novel and representative candidate for further validation.^49^, ^50^ Our analysis revealed that *PI4KB* exhibited elevated expression levels in tumor tissues compared to normal tissues (Figure S4). Meanwhile, previous studies indicate that upregulation of *PI4KB* contributes to tumor development and progression and it is recognized as a potential prognostic indicator in BC.^48^ To better understand the functional role of *PI4KB* in BC progression, we conducted multiple in vitro experiments focused on *PI4KB* silencing. Subsequently we examined the expression level of *PI4KB* in normal BC and *PI4KB* knockdown cell lines. Findings showed that *PI4KB* expression was down‐regulated in the knockdown group (Figure 8A). Knockdown of *PI4KB* inhibited BC cell proliferation in CCK‐8 and cell colony formation assays (Figure 8B,C). Wound healing and transwell assays showed that *PI4KB* knockdown inhibited BC cell invasion and migration (Figure 8D,E). Taken together, *PI4KB* plays a crucial role in the progression of BC.

## 4 DISCUSSION

Recent evidence increasingly supports the substantial therapeutic potential of paraptosis in anti‐cancer treatment.^51^ Paraptosis‐inducing agents have demonstrated considerable therapeutic potential when administered alone or in combination with standard chemotherapeutic agents, particularly in addressing chemoresistance.^15^, ^52^ Importantly, paraptosis also holds potential to enhance the efficacy of cancer immunotherapy.^22^, ^53^ Studies have demonstrated that the induction of paraptosis not only promotes tumor cell death but also remodels the TME and restores or activates immune function. These effects collectively contribute to enhanced therapeutic efficacy.^54^, ^55^ Altogether, these results underscore the potential of leveraging paraptosis as a novel therapeutic strategy in cancer treatment, although many challenges still remain before paraptosis‐based interventions can be successfully translated into clinical practice. Currently, this mechanism lacks clear biomarkers and specific signaling pathways, and paraptosis mainly relies on cytoplasmic vacuolization for recognition.^18^ Moreover, recent research has primarily examined paraptosis triggered by pharmacological agents, whereas its role under normal physiological and pathological conditions remains unclear.^9^ Some studies have shown that paraptosis can reshape the TME and affect immune cell function, but its specific role in immunotherapy and cellular immune mechanisms still needs to be further explored.^22^, ^49^ At present, the interaction between paraptosis and current cancer immunotherapies, including immune checkpoint inhibitors and other modalities, remains insufficiently studied. Future studies should focus on exploring the synergistic mechanism of paraptosis and immunotherapies to better elucidate its involvement in modulating the immune landscape.

We developed a risk scoring system to evaluate paraptosis in BC patients. This scoring model, referred to as PRRS, incorporates six genes: *PI4KB*, *HSPB8*, *USP10*, *LCK*, *GPR153*, and *NT5C*. The results indicate that PRRS serves as a robust prognostic biomarker for BC patients. OS was notably reduced in high‐risk individuals compared to those in the low‐risk group. Moreover, age, stage, and PRRS independently contributed to prognosis prediction. To explore the translational value of PRRS in clinical practice, a prognostic model combined with paraptosis‐related genes was used. The advanced ROC curve and calibration curve confirmed the high predictive accuracy of PRRS for BC prognosis.

In addition, various molecular characteristics were investigated among patients stratified into PRRS‐defined high‐ and low‐risk groups. DEGs between the two risk groups were significantly associated with various immune pathways. The majority of immune cells, including CD8+ T cells, Tregs, M1 macrophages, NK cells, and helper T cells in high‐risk patients were significantly less than those in low‐risk patients. A large proportion of immune‐associated genes showed lower expression levels in the high‐risk group, while the typical immune response in low‐risk patients was significantly enhanced. In addition, the low‐risk group also exhibited unique TME features such as elevated immune checkpoint expression and immune activation. Immune cells play a crucial role in mediating antitumor defense mechanisms, and the unfavorable prognosis observed in high‐risk individuals may stem from reduced immune cell presence and diminished immune defense capacity.^56^ Taken together, our study demonstrates that paraptosis may impact BC prognosis through its regulatory effects by modulating the immune microenvironment, suggesting that the low‐risk group may exhibit higher sensitivity to immunotherapy. Besides, drug sensitivity analysis showed that patients in the low‐risk group had a more sensitive response to CDK4/6 inhibitors (Palbociclib and Ribociclib), *PIK3CA* targeted drugs (Alpelisib), and estrogen receptor modulators (Epirubicin).

To enhance the reliability of our findings, we conducted in vitro assays to investigate the function of *PI4KB* in BC. *PI4KB* is a member of the *PI4K* family, which is implicated in the regulation of ER stress and autophagy.^57^ Previous research studies suggest that *PI4KB* plays a role in driving the progression of various BC subtypes and is closely associated with increased cancer cell growth, invasiveness, and metastatic potential. We verified that *PI4KB* is highly expressed in BC and found that downregulating its expression significantly suppressed tumor cell proliferation and motility.

This research study introduced the PRRS model for forecasting clinical outcomes and tumor microenvironment profiles in BC. Nevertheless, several limitations of this study should be acknowledged. Firstly, the clinical information obtained from public datasets such as TCGA and GEO has some incompleteness and limitations, which may have some impact on prognostic evaluation. However, given the large sample size in the training cohort and the good predictive capacity of the model when applied to an external validation cohort, the model still has high clinical practicability. Second, the reliability of this risk model needs to be further verified by large‐scale, prospective and multicenter studies in the future. Finally, our current study did not directly evaluate the role of individual paraptosis‐associated genes, such as PI4KB, in regulating paraptotic cell death. Although the functional assays demonstrated that PI4KB promotes proliferation and migration in BC cells, further mechanistic investigations are required to elucidate whether and how it contributes to paraptosis regulation. Future studies will aim to investigate the expression dynamics of PI4KB under classical paraptosis‐inducing conditions and to delineate its mechanistic relationship with ER stress, AKT/MAPK signaling, and autophagy regulation. These efforts will help clarify the functional relevance of paraptosis‐related genes and provide deeper insights into the molecular mechanisms driving the initiation and progression of BC.

## 5 CONCLUSION

In conclusion, this study identified *PI4KB*, *HSPB8*, *USP10*, *LCK*, *GPR153*, and *NT5C* as key biomarkers associated with paraptosis metabolism in BC. Our comprehensive analysis of the paraptosis‐related genes showed that it correlated with the prognosis and clinical features of BC as well as immune infiltration. These findings highlight the clinical importance of paraptosis‐related genes in predicting survival and guiding personalized treatment of BC. *PI4KB* may be a promising prognostic biomarker and therapeutic target, providing new insights into improving BC prognosis and treatment.

## AUTHOR CONTRIBUTIONS

Ziyi Dong and Yanfang Yang contributed to the methodology, data acquisition, and analysis. Mingyu Zhu, Yaoyang Guo and Hui Liu drafted the manuscript. Haiyang Zhang and Zhansheng Jiang conceived the study. All authors have read and agreed to this version of the manuscript. All authors contributed to the article and approved the submitted version.

## CONFLICT OF INTEREST STATEMENT

The authors declare no conflicts of interest.

## ETHICS STATEMENT

Not applicable.

## Supporting information

Figures S1–S4

Table S1

Table S2

Table S3

Table S4

## Data Availability

The data generated and analyzed during the current study are available from the corresponding author upon reasonable request.
