# Supplementary material for: Paraptosis‐related genes regulate tumor immune microenvironment and predict prognosis in breast cancer
Source: J Cell Commun Signal. 2025 Dec 8;19(4):e70056. doi: 10.1002/ccs3.70056 (PMC12685561; doi:10.1002/ccs3.70056)
Supplement: Supplementary file 1 — Figures S1–S4 [file CCS3-19-e70056-s005.docx]

**
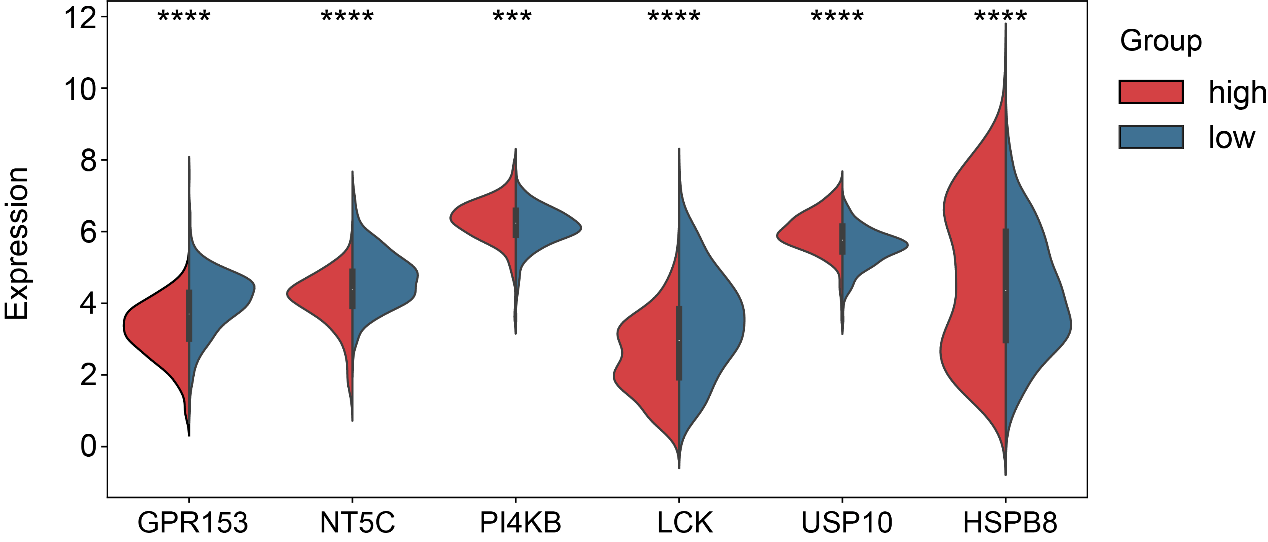
**

**Fig. S1** Differential expression of the six key genes in the high and low risk groups. (****p* < 0.001; *****p* < 0.0001)


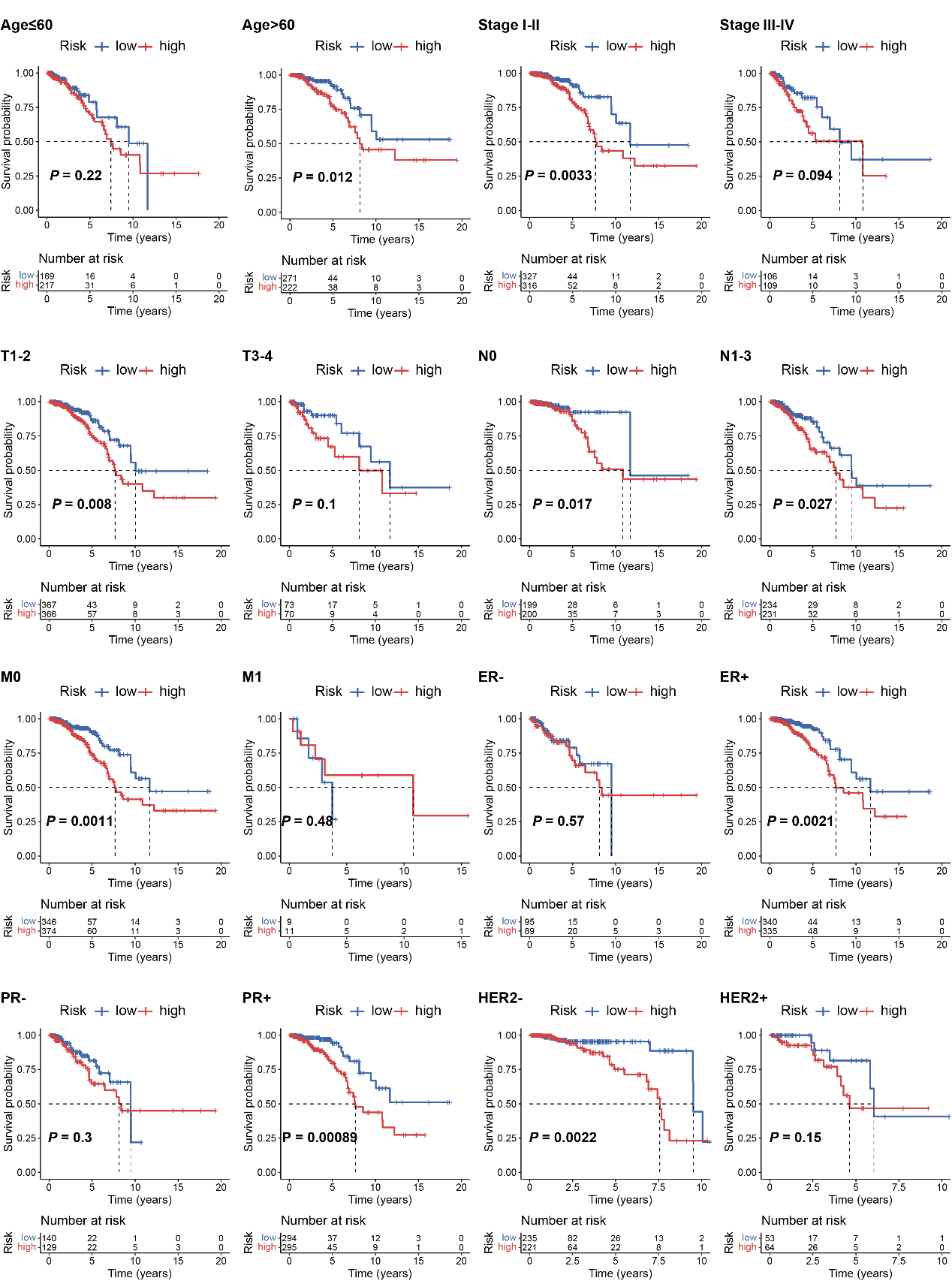


**Fig. S2** Kaplan–Meier survival analysis for predicting survival in BC training patients with different clinical features

**
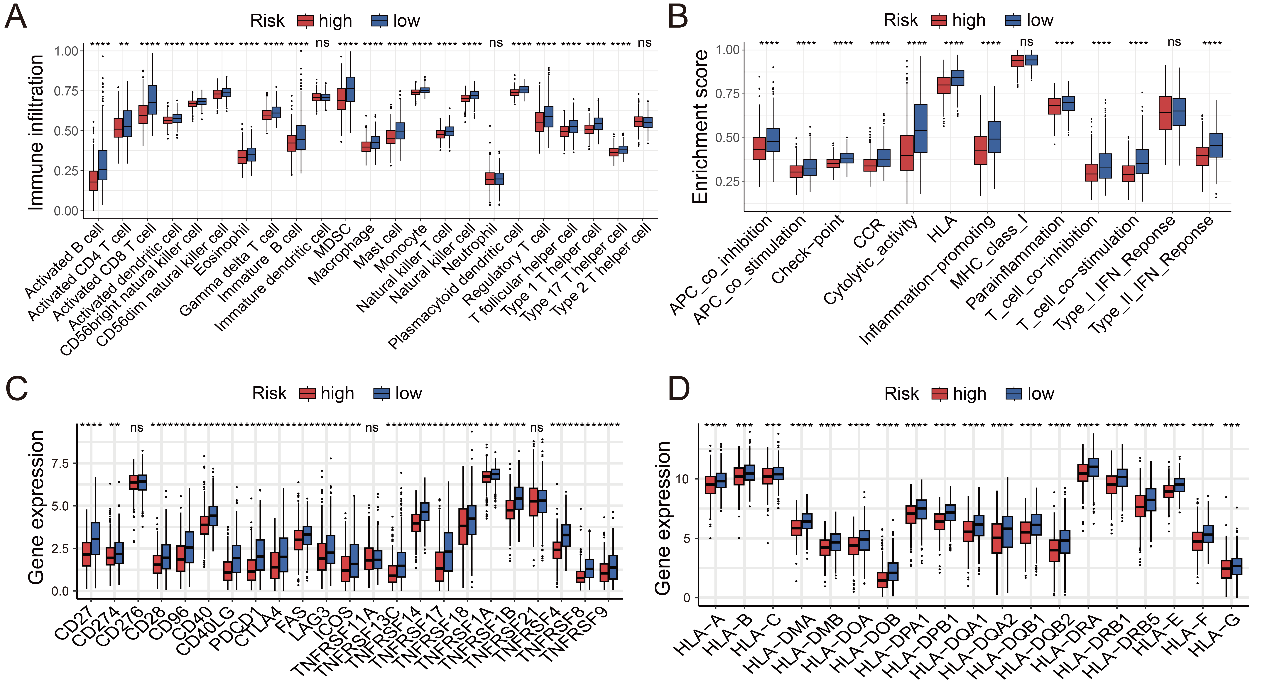
**

**Fig. S3** Immune landscape between the two PRRS groups. A, B The analysis of differences in immune cell infiltration and function between the two groups with ssGSEA. C, D Comparison of expression levels of immune-related genes between the two subgroups. (***p* < 0.01; ****p* < 0.001; *****p* < 0.0001; ns, not significant)


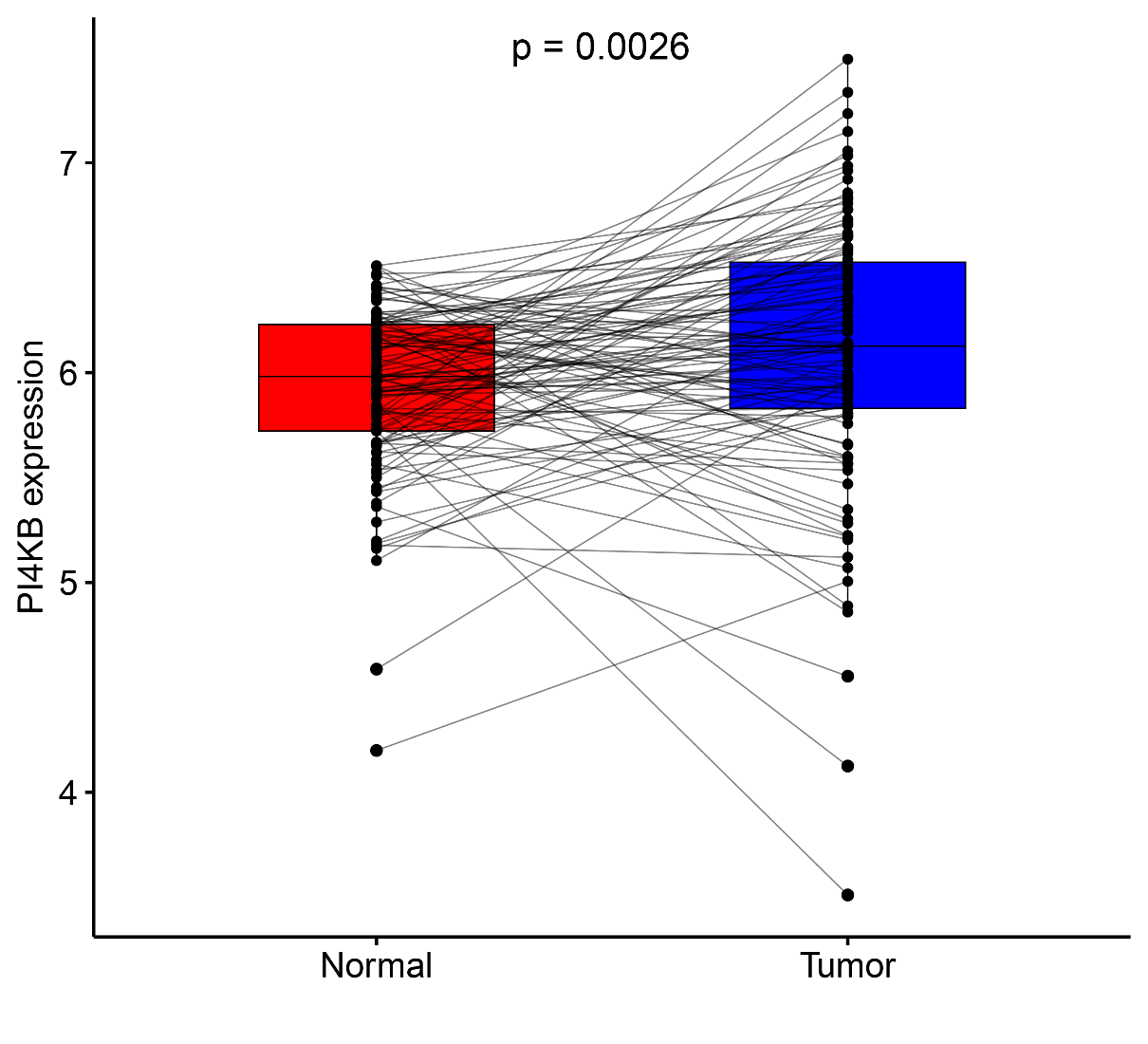


**Fig. S4** The expression of *PI4KB* in tumor and normal
